# 3-D magnetohydrodynamic AA7072-AA7075/methanol hybrid nanofluid flow above an uneven thickness surface with slip effect

**DOI:** 10.1038/s41598-020-61215-8

**Published:** 2020-03-06

**Authors:** Iskander Tlili, Hossam A. Nabwey, G. P. Ashwinkumar, N. Sandeep

**Affiliations:** 1 Department for Management of Science and Technology Development, Ton Duc Thang University, Ho Chi Minh City, Vietnam; 2 Faculty of Applied Sciences, Ton Duc Thang University, Ho Chi Minh City, Vietnam; 3 Department of Mathematics, College of Science and Humanities in Al-Kharj, Prince Sattam bin Abdulaziz University, Al-Kharj, 11942 Saudi Arabia; 4 Department of Basic Engineering Science, Faculty of Engineering, Menoufia University, Shebin El-Kom, 32511 Egypt; 5 Department of Mathematics, Vijayanagara Sri Krishnadevaraya University, Ballary, 583105 India; 6 Department of Mathematics, Central University of Karnataka, Kalaburagi, 585 367 India

**Keywords:** Applied mathematics, Fluid dynamics

## Abstract

A 3-D magnetohydrodynamic flow of hybrid nanofluid across a stretched plane of non-uniform thickness with slip effects is studied. We pondered aluminum alloys of AA7072 and AA7072 + AA7075 in methanol liquid. The aluminum alloys amalgamated in this study are uniquely manufactured materials, possessing enhanced heat transfer features. AA7072 alloy is a composite mixture of Aluminum & Zinc in the ratio 98 & 1 respectively with added metals Silicon, ferrous and Copper. Equally, AA7075 is a mixture of Aluminum, Zinc, Magnesium, and Copper in the ratio of ~90, ~6, ~3 and ~1 respectively with added metals Silicon ferrous and Magnesium. Numerical solutions are attained using R-K based shooting scheme. Role of physical factors on the flow phenomenon are analyzed and reflected by plots and numerical interpretations. Results ascertain that heat transfer rate of the hybrid nanoliquid is considerably large as matched by the nanofluid. The impact of Lorentz force is less on hybrid nanofluid when equated with nanofluid. Also, the wall thickness parameter tends to improve the Nusselt number of both the solutions.

## Introduction

Advanced electronic gadgets frequently encounter challenges because of heat control from enhanced thermal rise or reduction of available space for the thermal emission. Such drawbacks are overwhelmed by developing a preeminent model for heat-repelling gadgets or by amplifying thermal transport features. Nanofluid is a unique and well-suited fluid to fit for all needs. Initially, Choi^1^ has experimented on the treatment of solid particles in conventional liquids to improve its thermal performance characterized as nanoliquid. Due to its marvelous thermal and chemical properties, less volume and enhanced thermal properties, it is emerging as an extensively used cooling agent. Nanofluid has entered in many areas of science and engineering, and few are witnessed in nuclear cooling, biomedical applications, electronic cooling, etc. Because of its massive demand, it has attracted the research community to develop a new class of nanofluids. Few researchers (^2–11^) provided the theoretical and experimental studies for developing nanofluids in terms of preparation methods, applications and enhancing its thermal properties. Further, Animasaun *et al*.^12^ deliberated the comparative study for distinct magnitude aluminum nanomaterials suspended in water, namely, 36 nanometers and 47 nanometers and predicted that 36 nm nanoparticle used to attain maximum flow velocity than other. Asadi *et al*.^13^ explained the flow of nanofluid (10 nanometer-sized Fe_3_O_4_ nanoparticles) across a sinusoidal crumpled section accounting the magnetic field effects. Later, Kumar *et al*.^14^ elaborated the stagnated flow caused by non-Newtonian liquids over a strained cylinder using C-C heat flux model. They concluded that friction factor parameter hikes significantly in Williamson liquid as compared with Casson liquid under the influence of thermal relaxation parameter. This kind of work was prolonged by Bai *et al*.^15^ using Oldroyd-B nanofluid.

MHD describes the magnetic properties of electrically conducting fluids. Theoretical investigation on CNT-water nanoliquid motion through a rectangular region using Hamilton-Crosser model was scrutinized by Benos *et al*.^16^. They noticed that variation in the shape of the nanomaterials tends to enhance heat transfer performance. Moreover, Chamkha^17^ discussed numerically the impression of magnetic properties over the nano liquid flow caused due cylinder in a three-dimensional enclosure by the aid of the finite element method. Meanwhile, the joint response of Prandtl number and magnetic properties over the 2D steady motion of nanofluid past a stretched membrane was numerically explored by Ganesh *et al*.^18^. As per the available literature, several researchers (^19–23^) did the outstanding work on applying MHD concept in their analysis.

Radiative thermal emission found vital applications in industrial engineering as the construction of gas turbines, design of fin and missiles, etc. Khan *et al*.^24^ deliberated the 2-D flow of nano liquid through melting plane under the response of radiative heat flux^24^. They revealed that hike in thermal radiation results in the improvement of heat transfer performance of the liquid. Seth *et al*.^25^ examined semi analytically with the aid of OHAM to study the flow of nanofluid through an elongated plane by the implication of magnetic properties and also examined the entropy generation. Further, the researchers^26–31^ made noticeable results their analysis in convective heat transfer. Acharya *et al*.^32^ explored a computational work for analyzing the multiple slip effects on chemically reacting Williamson fluid flow in permeable medium. A hybrid approach for investigating the thermal radiation and hall current effects on nanoliquid flow over a spinning disk was proposed by Acharya *et al*.^33^. The effect of aligned magnetic field on the slippery flow of nanofluid was numerically studied by Acharya *et al*.^34^. The researchers^35,36^ investigated the convective heat transport in different nanofluids using NDM and Lie group approaches. Effect of internal heat source and radiation on 3-D flow of nanofluid past a shrinking sheet was theoretically studied by Sharma *et al*.^37^. The researchers^38,39^ investigated the natural convection in magnetohydrodynamic flow under various physical effects. Thermal radiation effect on magnetohydrodynamic flow in the presence of heat generation was numerically studied by the researchers^40,41^. Boling *et al*.^42^ proposed a stability solution for the MHD equation. The researchers^43,44^ studied the magnetohydrodynamic flow of Power-Law fluid by considering the various flow geometries. Recently, Tlili *et al*.^45,46^ premeditated the magnetohydrodynamic flow of nanofluid by considering the various physical effects and flow geometries.

Recent days, variety of nanomaterial are discovered in literature, among these aluminum alloy nanoparticles AA7075 and AA7072 are of special featured nanomaterial with greater thermal, chemical and physical properties. Aluminum alloy plays a prominent role in aerospace industries, especially, aluminum alloys AA7072 and AA7075 are of abundant significance in the production of transport appliances namely, glider aircraft, rocket climbing frame, etc.^29^. It is evident that very less work has found in the study of hybrid nanofluids. This article reports the 3-D magnetohydrodynamic flow of hybrid nanofluid across a stretched plane of non-uniform thickness with slip effects. We pondered aluminum alloys of AA7072 and AA7072 + AA7075 in methanol liquid. The numerical solutions are attained, and the role of physical factors on the flow phenomenon is analyzed and reflected by plots and numerical interpretations.

## Formulation

3D MHD, steady flow of hybrid nanofluid past a stretched plane of non-uniform thickness with slip effect is considered. The hybrid nanofluid is composed of alloy nanoparticles of AA7072 and AA7072 + AA7075 suspended in methanol liquid.

**Figure 1:**
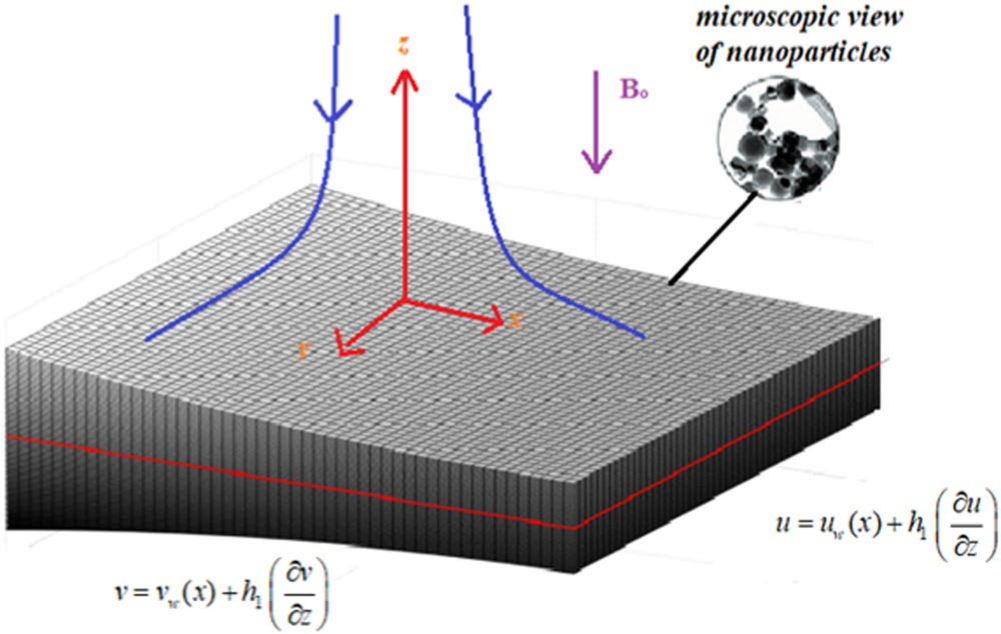
Schematic Model.

The sheet of non-uniform thickness is considered as $z=A{\delta }^{(1-n)/2},\,\delta =x+y+c,n\ne 1$ we have chosen *A* is small. It is also presumed, the sheet temperature as ${T}_{w}={T}_{0}{\delta }^{\frac{1-n}{2}}+{T}_{\infty }$. The induced magnetic field is ignored in this study. Here *B*_0_ is the magnetic field applied in parallel with the *z*− axis as revealed in Fig. 1. With conventions made above, the governing equations in vector form can be expressed as^30^:

1 $$\nabla .q=0,$$

2 $${\rho }_{hnf}(q.(\nabla u))={\mu }_{hnf}{\nabla }^{2}u-{\sigma }_{hnf}{B}^{2}u,$$

3 $${\rho }_{hnf}(q.(\nabla v))={\mu }_{hnf}{\nabla }^{2}v-{\sigma }_{hnf}{B}^{2}v,$$

4 $${(\rho {c}_{p})}_{hnf}(q.(\nabla T))={k}_{hnf}{\nabla }^{2}T,$$

the linked boundary restrictions are

5 $$\begin{array}{c}u-{u}_{w}(x)-{h}_{1}(\frac{\partial u}{\partial z})=0,v-{v}_{w}(x)-{h}_{1}(\frac{\partial v}{\partial z})=0,\\ T-{T}_{w}(x)-{h}_{2}(\frac{\partial T}{\partial z})=0,\\ {\rm{and}}\,u,v\to 0,\,T\to {T}_{\infty }\,{\rm{as}}\,{\rm{z}}\to \infty \end{array}\}$$

where

6 $$\begin{array}{c}{\zeta }_{1}=\frac{{k}_{B}T}{\sqrt{2}\pi {d}^{2}p},\delta =x+y+c,\,{h}_{1}=[\frac{2-{f}_{1}}{{f}_{1}}]{\zeta }_{1}{\delta }^{\frac{1-n}{2}},\\ {\zeta }_{2}=(\frac{2\gamma }{\gamma +1})\frac{{\zeta }_{1}}{\Pr },{h}_{2}=[\frac{2-b}{b}]{\zeta }_{2}\,{\delta }^{\frac{1-n}{2}},\,B={B}_{0}{\delta }^{0.5(n-1)},\end{array}\}$$

7 $${u}_{w}=a{\delta }^{(1/2)(n-1)},\,{v}_{w}=a{\delta }^{n},\,{T}_{w}-{T}_{\infty }={T}_{0}\,{\delta }^{\frac{1-n}{2}},\,{\rm{for}}\,n\ne 1,$$

The hybrid nanofluid parameters ${\rho }_{nf},{\mu }_{nf},{\sigma }_{nf},{k}_{nf}$ represent the density, dynamic viscosity, electrical conductivity, thermal conductivity can be used as^26^:

8 $$\begin{array}{c}\frac{{k}_{hnf}}{{k}_{f}}=\frac{2(1-\phi ){k}_{f}+(1+2{\phi }_{1s}){k}_{1s}+(1+2{\phi }_{2s}){k}_{2s}}{(2+\phi ){k}_{f}+(1-{\phi }_{1}){k}_{1s}+(1-{\phi }_{2}){k}_{2s}},\\ \frac{{\rho }_{hnf}}{{\rho }_{f}}=(1-\phi )+\frac{{\phi }_{1s}{\rho }_{1s}}{{\rho }_{f}}+\frac{{\phi }_{2s}{\rho }_{2s}}{{\rho }_{f}},\frac{{(\rho {c}_{p})}_{hnf}}{{(\rho {c}_{p})}_{f}}=(1-\phi )+\frac{{\phi }_{1s}{(\rho {c}_{p})}_{1s}+{\phi }_{2s}{(\rho {c}_{p})}_{2s}}{{(\rho {c}_{p})}_{f}},\\ \frac{{\mu }_{hnf}}{{\mu }_{f}}={(1-\phi )}^{-2.5},\frac{{\sigma }_{hnf}}{{\sigma }_{f}}=[1+\frac{3{\sigma }_{1s}{\phi }_{1s}+{\phi }_{2s}{\sigma }_{2s}-3\phi {\sigma }_{f}}{{\sigma }_{1s}(1-{\phi }_{1s})+{\sigma }_{2s}(1-{\phi }_{2s})+(2+\phi ){\sigma }_{f}}],\phi ={\phi }_{1s}+{\phi }_{2s},\end{array}\}$$

following similarity transformations are used for non-dimensionalisation

9 $$\begin{array}{c}\eta =z{(\frac{(n+1)a}{2\upsilon })}^{1/2}{\delta }^{(n-1)/2},T-{T}_{\infty }-({T}_{w}(x)-{T}_{\infty })\theta =0,\\ u-a{\delta }^{n}f{\prime} (\eta )=0,v-a{\delta }^{n}g{\prime} (\eta )=0,\\ w=-{(\frac{2a\nu }{n+1})}^{0.5}{\delta }^{(n-1)0.5}[\frac{n+1}{2}(f+g)+\eta (\frac{n-1}{2})(f{\prime} +g{\prime} )]\end{array}\}$$

by making use of Eqs. (6–9), the Eqs. (1–5) can be transmuted as

10 $$\begin{array}{c}\frac{n+1}{2{(1-\phi )}^{2.5}}f{\prime} {\prime} {\prime} -((1-\phi )+\frac{{\phi }_{1s}{\rho }_{1s}+{\phi }_{2s}{\rho }_{2s}}{{\rho }_{f}})(n{(f{\prime} )}^{2}+nf{\prime} g{\prime} -\frac{n+1}{2}(f+g)f{\prime\prime} )\\ \,-(1+\frac{3{\sigma }_{1s}{\phi }_{1s}+{\phi }_{2s}{\sigma }_{2s}-3\phi {\sigma }_{f}}{{\sigma }_{1s}(1-{\phi }_{1s})+{\sigma }_{2s}(1-{\phi }_{2s})+(2+\phi ){\sigma }_{f}})Mf{\prime} =0,\end{array}\}$$

11 $$\begin{array}{c}\frac{n+1}{2{(1-\phi )}^{2.5}}g{\prime} {\prime} {\prime} -((1-\phi )+\frac{{\phi }_{1s}{\rho }_{1s}+{\phi }_{2s}{\rho }_{2s}}{{\rho }_{f}})(n{(g\text{'})}^{2}+nf{\prime} g{\prime} -\frac{n+1}{2}(f+g)g{\prime\prime} )\\ \,-(1+\frac{3{\sigma }_{1s}{\phi }_{1s}+{\phi }_{2s}{\sigma }_{2s}-3\phi {\sigma }_{f}}{{\sigma }_{1s}(1-{\phi }_{1s})+{\sigma }_{2s}(1-{\phi }_{2s})+(2+\phi ){\sigma }_{f}})Mg{\prime} =0,\end{array}\}$$

12 $$\begin{array}{c}(\frac{2(1-\phi ){k}_{f}+(1+2{\phi }_{1s}){k}_{1s}+(1+2{\phi }_{2s}){k}_{2s}}{(2+\phi ){k}_{f}+(1-{\phi }_{1}){k}_{1s}+(1-{\phi }_{2}){k}_{2s}})\theta {\prime\prime} -\frac{2\Pr }{n+1}((1-\phi )+\frac{{\phi }_{1s}{(\rho {c}_{p})}_{1s}+{\phi }_{2s}{(\rho {c}_{p})}_{2s}}{{(\rho {c}_{p})}_{f}})\\ \,(\frac{1-n}{2}\,\theta \,(f{\prime} +g{\prime} )-\frac{n+1}{2}\theta {\prime} (f+g))=0,\end{array}\}$$

the transmuted boundary restrictions are

13 $$\begin{array}{c}f(0)=\Lambda (\frac{1-n}{n+1})[1+{h}_{1}f\text{'}\text{'}{(\eta )}_{\eta =0}],f{\prime} (0)=[1+{h}_{1}\,f{\prime\prime} {(\eta )}_{\eta =0}],\\ g(0)=\Lambda (\frac{1-n}{n+1})[1+{h}_{1}g{\prime\prime} {(\eta )}_{\eta =0}],\theta (0)=[1+{h}_{2}\theta {\prime} (0)],\\ g{\prime} (0)=[1+{h}_{1}g{\prime\prime} (0)],f{\prime} {(\eta )}_{\eta \to \infty }=0,g{\prime} {(\eta )}_{\eta \to \infty }=0,\theta {(\eta )}_{\eta \to \infty }=0,\end{array}\}$$

where

14 $$M=\frac{{\sigma }_{f}{B}_{0}^{2}}{{\rho }_{f}a},\Pr =\frac{{\mu }_{f}{({c}_{p})}_{f}}{{k}_{f}},\Lambda =\frac{1}{{(1-\phi )}^{2.5}}\sqrt{\frac{(n+1)a}{2\upsilon }},$$

are the magnetic field parameter, Prandtl number and wall thickness parameters respectively. For engineering curiosity the *C*_*f*_ and *Nu*_*x*_ are defined as

15 $${C}_{f}=2\frac{{\mu }_{hnf}}{{\mu }_{f}\sqrt{\mathrm{Re}}}{(\frac{n+1}{2})}^{0.5}{f{\prime\prime} |}_{\eta =0},\,N{u}_{x}=-\sqrt{\mathrm{Re}}\frac{{k}_{hnf}}{{k}_{f}}{(\frac{n+1}{2})}^{0.5}{\theta {\prime} |}_{\eta =0}\}$$

where $$\mathrm{Re}=\frac{{u}_{w}\delta }{{\upsilon }_{f}},$$

## Results and Discussion

The system of ODE’s (10–12) along the boundary restrictions (13) are resolved numerically using R-K based shooting procedure^14^. Impression of diverse dimensionless factors, volume fraction (*ϕ*), magnetic field (*M*), velocity power index (*n*), velocity slip (*h*_1_), temperature jump (*h*_2_), and wall thickness (Λ) over common profiles are revealed with plots and the influence of same restrictions on $f{\prime\prime} (0)$ and $-\theta {\prime} (0)$ are depicted in a tabular manner. The physical parametric values are set to $M=1,n=0.7,{h}_{1}=0.4,{h}_{2}=0.4,\Lambda =0.1,\Pr =7.38$ in order to attain the required results. Above quantities are reserved for the complete study, unless they specified in respective graphs and tables. Symbols used in figures $f{\prime} (\eta )$, $g{\prime} (\eta )$ and *θ*(*η*) describes the flow common quantities as velocity and temperature respectively. Simultaneous solutions are noticed for Methanol+AA7075 nanofluid and Methanol+AA7075 + AA7072 nanofluid. We treat Methanol+AA7075 nanofluid as first solution and Methanol+AA7075 + AA7072 nanofluid as second solution.

**Figure 2:**
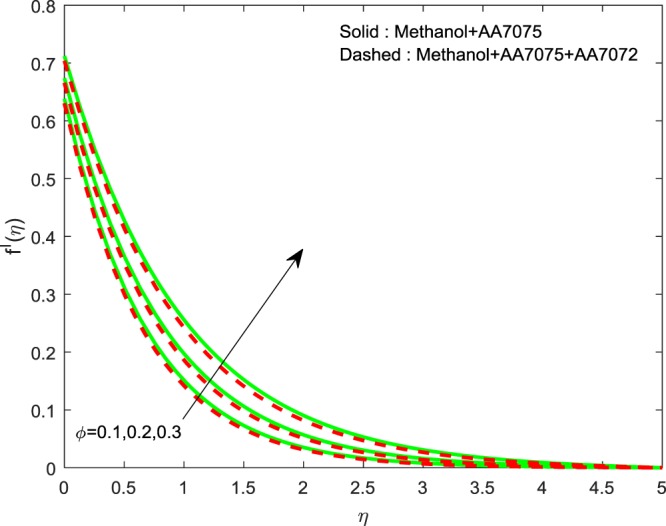
Impression of *ϕ* on $f{\prime} (\eta )$.

**Figure 3:**
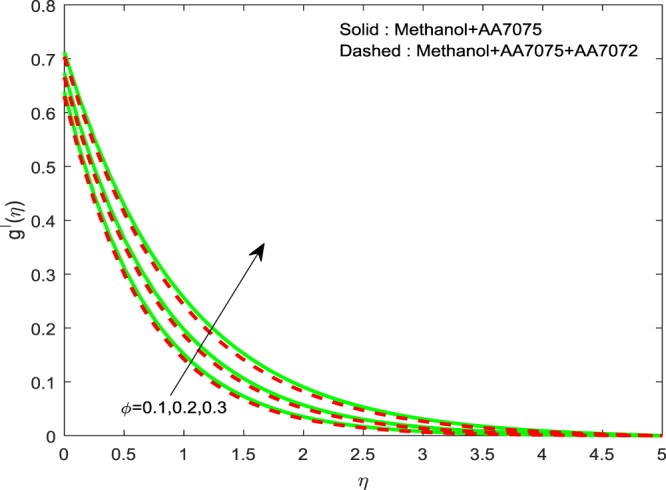
Impression of *ϕ* on $g{\prime} (\eta )$.

**Figure 4:**
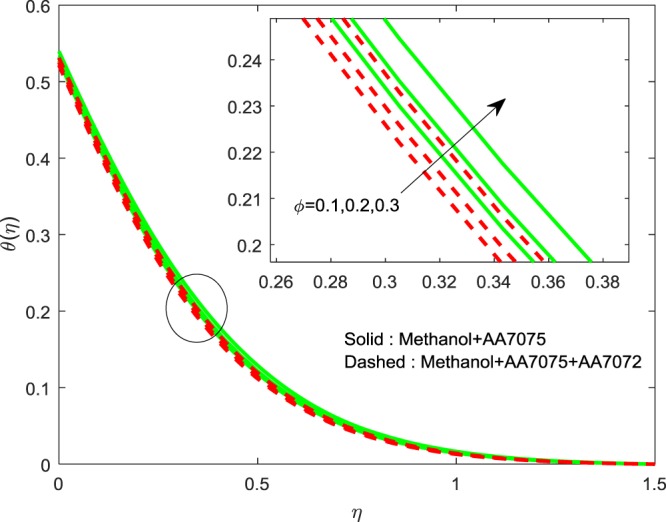
Impression of *ϕ* on $\theta (\eta )$.

Figures 2–4 exhibits the impact of (*ϕ*) on $f{\prime} (\eta )$, $g{\prime} (\eta )$ and *θ*(*η*) we detect a hike in $f{\prime} (\eta )$, $g{\prime} (\eta )$ and *θ*(*η*) for improvement in volume of (*ϕ*). The methanol+AA7075 nanofluid flow is highly influenced for rise in (*ϕ*) than Methanol+AA7075 + AA7072 nanofluid. Physically, rising the nanoparticle volume fraction leads to enhance the thermal conductivity of the fluid.

**Figure 5:**
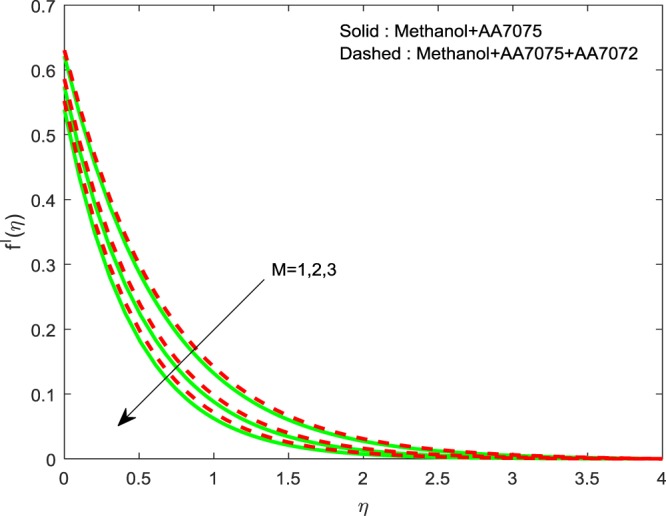
Impression of *M* on $f{\prime} (\eta )$.

**Figure 6:**
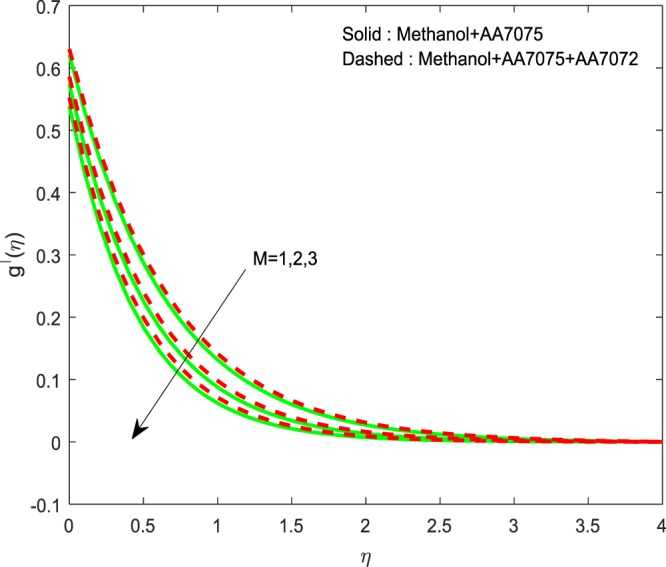
Impression of *M* on $g{\prime} (\eta )$.

**Figure 7:**
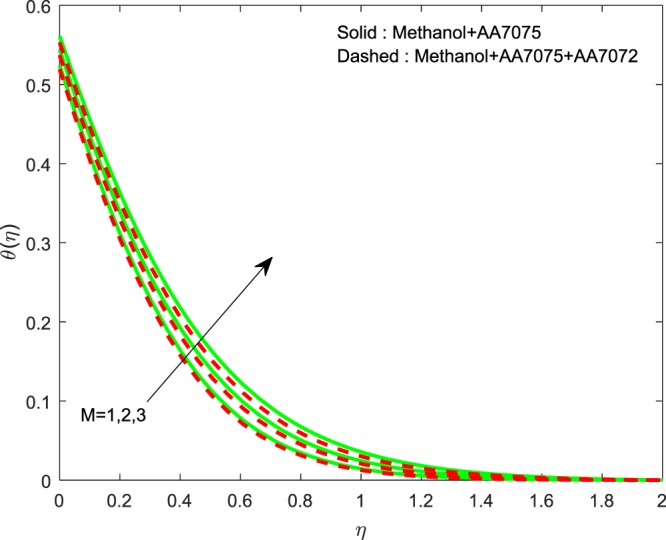
Impression of *M* on $\theta (\eta )$.

Figures 5–7 depicted to witness the effect of Lorentz force on $f{\prime} (\eta )$, $g{\prime} (\eta )$ and *θ*(*η*). We conclude that, increase in *M* upshots the reduction of $f{\prime} (\eta )$ and $g{\prime} (\eta )$. And a reverse trend is detected for *θ*(*η*). Physically, improvement in *M* leads to develop Lorentz force which in turn causes to resist the fluid motion, hence, we notice upswing in thermal boundary layer. The existence of *M* diminishes the fluid motion of Methanol+AA7075 + AA7072 nanoliquid over the Methanol+AA7075 nanoliquid

**Figure 8:**
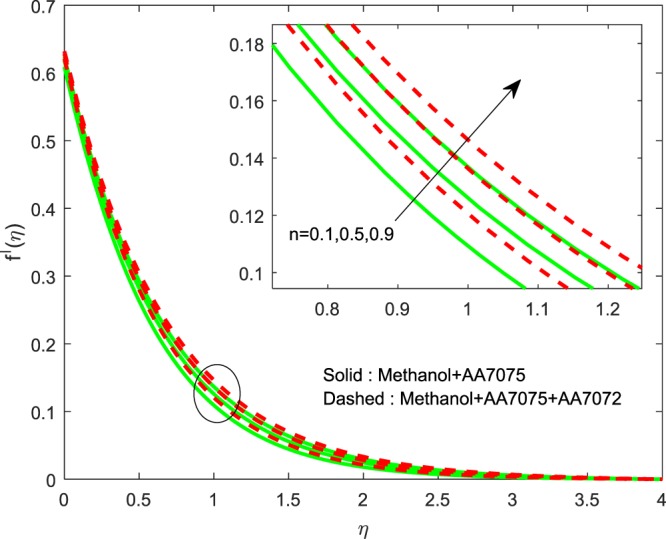
Impression of *n* on $f{\prime} (\eta )$.

**Figure 9:**
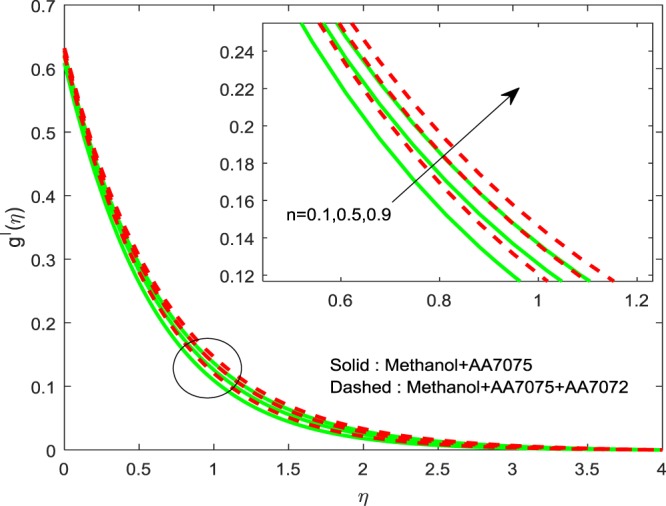
Impression of *n* on $g{\prime} (\eta )$.

**Figure 10:**
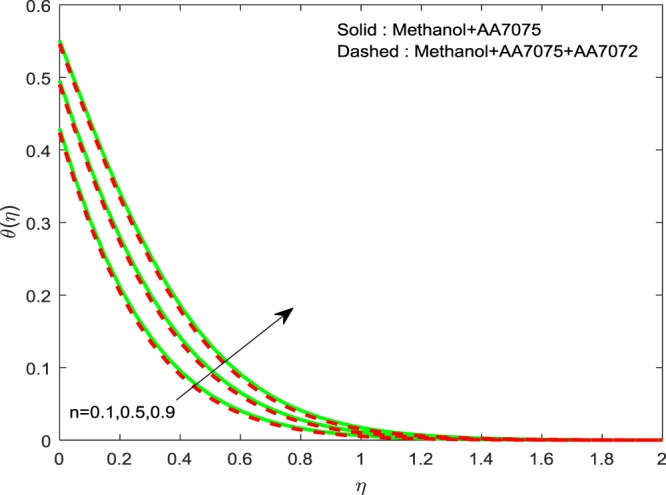
Impression of *n* on $\theta (\eta )$.

**Figure 11:**
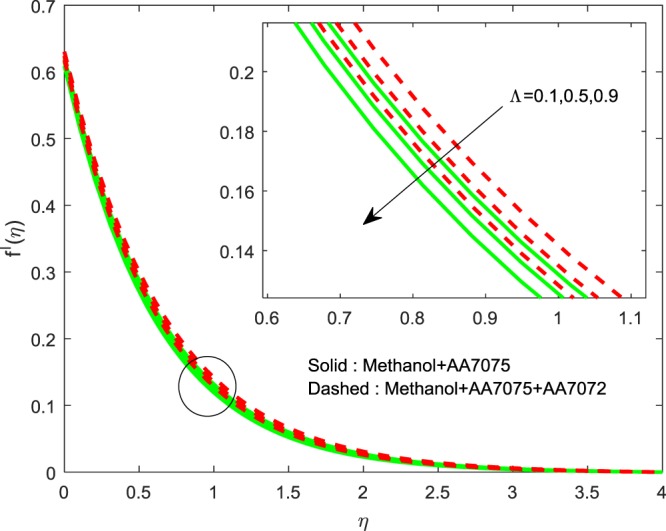
Impression of Λ on $f{\prime} (\eta )$.

**Figure 12:**
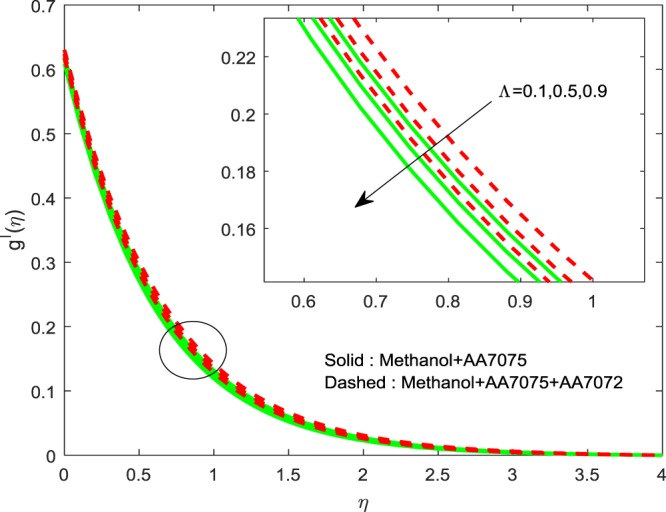
Impression of Λ on $g{\prime} (\eta )$.

**Figure 13:**
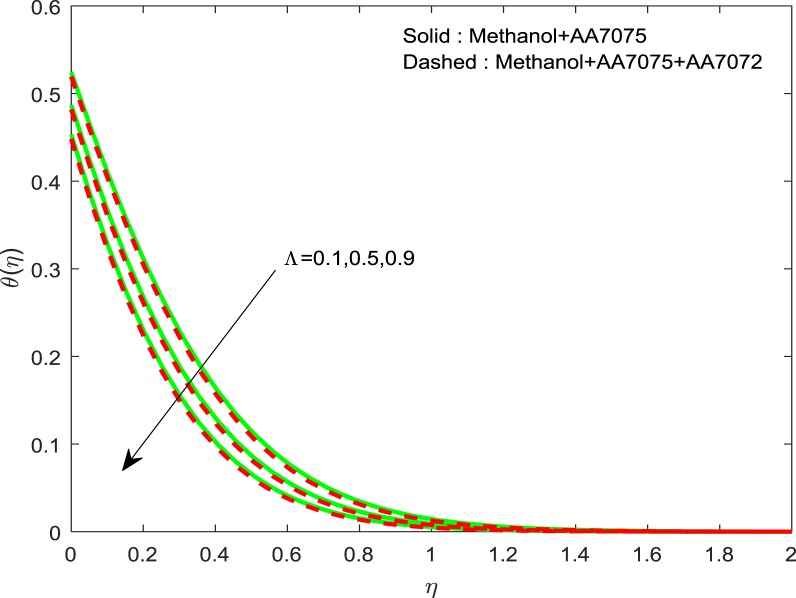
Impression of Λ on $\theta (\eta )$.

Figures 8–10 are depicted to ascertain the nature of the curvatures $f{\prime} (\eta )$, $g{\prime} (\eta )$ and *θ*(*η*) under the influence of $n$. It is clear that, rise in $n$ improves the distributions for $f{\prime} (\eta )$, $g{\prime} (\eta )$ and *θ*(*η*). Actually, boosting *n* helps in slendering of the sheet. It leads to, weaken the thickness of the sheet and in turn it enhances the thermal boundary layers. Figures 11–13 outlined to witness the consequences of Λ on $f{\prime} (\eta )$, $g{\prime} (\eta )$ and *θ*(*η*). We found that, $f{\prime} (\eta )$, $g{\prime} (\eta )$ and *θ*(*η*) are decreasing function of Λ. This concur the physical nature of the wall thickness parameter.

**Figure 14:**
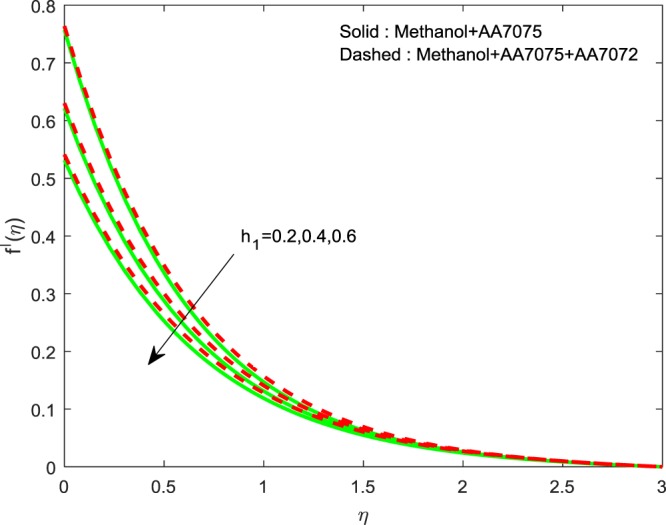
Impression of *h*_1_ on $f{\prime} (\eta )$.

**Figure 15:**
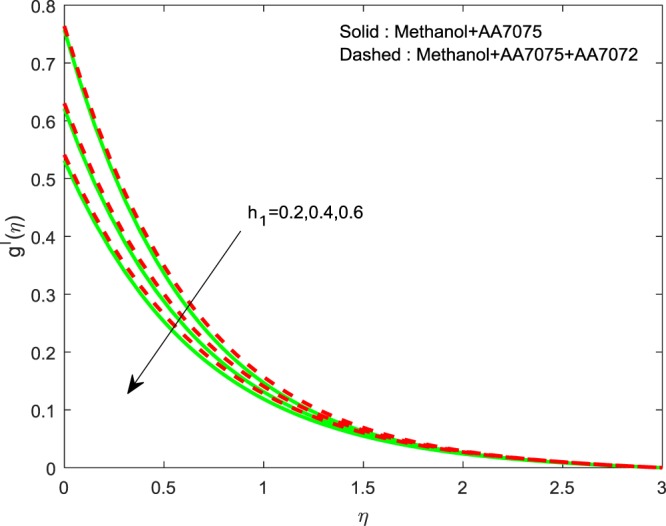
Impression of *h*_1_ on $g{\prime} (\eta )$.

**Figure 16:**
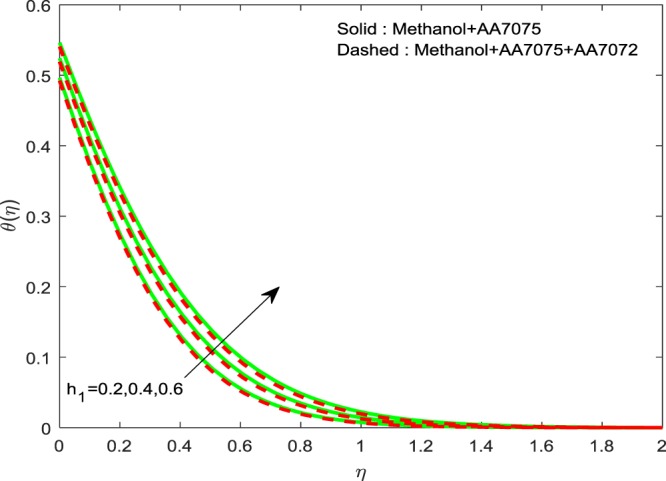
Impression of *h*_1_ on $\theta (\eta )$.

**Figure 17:**
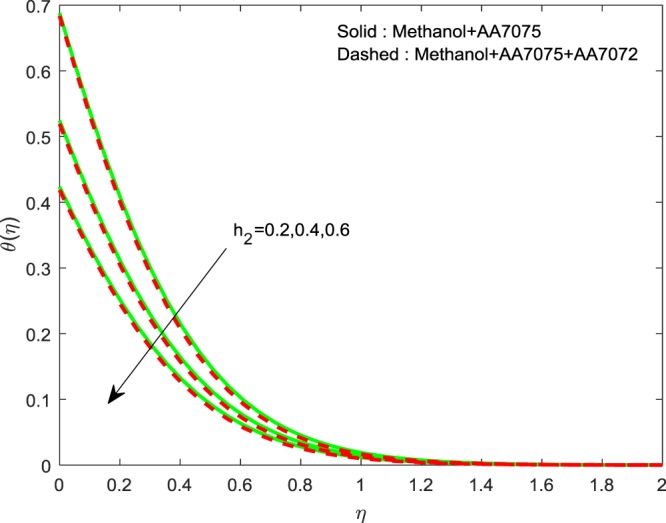
Impression of *h*_2_ on $\theta (\eta )$.

Figures 14–16 are portrayed to witness the changes in $f{\prime} (\eta )$, $g{\prime} (\eta )$ and *θ*(*η*) for diverse values of *h*_1_. It is evident that, escalating values of *h*_1_ improves *θ*(*η*), but reverse nature is observed for $f{\prime} (\eta )$ and $g{\prime} (\eta )$. Finally, Fig. 17 exhibits the impact of *h*_2_ on *θ*(*η*). It is obvious that, temperature distributions are diminishing functions of *h*_2_.

Table 1 portrays the basic properties of base liquid and nanoscaled materials. The disparity in skin friction factor $f{\prime\prime} (0)$ and Nusselt number $-\theta {\prime} (0)$ under the influence of flow parameters $\phi ,M,n,\Lambda ,{h}_{1}\,{\rm{and}}\,{h}_{2}$ are depicted in Table 2. The following observations are made, improved values of *M* and *n* results in declination of both skin friction coefficient and rate of heat transfer. It also worth noting that, the values $f{\prime\prime} (0)$ and $-\theta {\prime} (0)$ of methanol+AA7072 + AA7075 nanofluid are more influenced by the varied values of *M* and *n* when compared with methanol+AA7075 nanofluid. Rate of heat transfer is a rising function of Λ, and $-\theta {\prime} (0)$ of methanol+AA7072 + AA7075 solution is high as equated with methanol+AA7075 solution. They are intensifying the values of *ϕ* and *h*_1_, both the parameters $f{\prime\prime} (0)$ and $-\theta {\prime} (0)$ decelerates. Thermal transport rate of the nanofluids diminishes for improved vales of *h*_2_. The validation of the present results is depicted in Table 3.

**Table 1:** Physio-thermal properties^29^.

Thermo Physical Properties	Methanol	AA7075	AA7072
*ρ*(Kg/m^3^)	792	2810	2720
*c*_*p*_(JKg^−1^ K^−1^)	2545	960	893
*k*(Wm^−1^ K^−1^)	0.2035	173	222
σ (S/m)	0.5 × 10^−6^	26.77 × 10^6^	34.83 × 10^6^

**Table 2:** Values of $f{\prime\prime} (0)$ and $-\theta {\prime} (0)$ for diverse non-dimensional constraints.

	*ϕ*	*M*	*n*	Λ	*h*_1_	*h*_2_	$f{\prime\prime} (0)$	$-\theta {\prime} (0)$
Methanol+AA7075	0.1						−0.904541	1.189994
	0.2						−0.815286	1.183189
	0.3						−0.719774	1.171804
Methanol+AA7075 + AA7072	0.1						−0.924201	1.204535
	0.2						−0.835555	1.200096
	0.3						−0.740353	1.191678
Methanol+AA7075		1					−0.946854	1.191963
		2					−1.064841	1.140875
		3					−1.153017	1.096588
Methanol+AA7075 + AA7072		1					−0.924250	1.204508
		2					−1.035379	1.158209
		3					−1.119866	1.117809
Methanol+AA7075			0.1				−0.977908	1.427021
			0.5				−0.954347	1.263593
			0.9				−0.940993	1.125571
Methanol+AA7075 + AA7072			0.1				−0.947767	1.441625
			0.5				−0.929845	1.276739
			0.9				−0.919909	1.137567
Methanol+AA7075				0.1			−0.946854	1.191963
				0.5			−0.962395	1.285095
				0.9			−0.977748	1.368908
Methanol+AA7075 + AA7072				0.1			−0.924250	1.204508
				0.5			−0.939929	1.297872
				0.9			−0.955430	1.381794
Methanol+AA7075					0.2		−1.214595	1.260707
					0.4		−0.947198	1.191768
					0.6		−0.781192	1.137189
Methanol+AA7075 + AA7072					0.2		−1.180789	1.270845
					0.4		−0.924716	1.204259
					0.6		−0.764753	1.151438
Methanol+AA7075						0.2	−0.951100	1.561062
						0.4	−0.951100	1.189641
						0.6	−0.951100	0.960994
Methanol+AA7075 + AA7072						0.2	−0.929448	1.582044
						0.4	−0.929448	1.201788
						0.6	−0.929448	0.968904

**Table 3:** Validation of the results for $f{\prime\prime} (0)$ (2D case-water with ϕ = 0) for various values of Λ and *h*_1_.

*h*_1_	Λ	ref. ^31^	Present Results
0	0.2	−0.924828	−0.924828342
0.2	0.25	−0.733395	−0.733395213
0.2	0.5	−0.759570	−0.759570103

## Conclusions

A 3D MHD flow of hybrid nanofluid over a surface of non-uniform thickness with slip effects is studied numerically. We pondered aluminum alloys of AA7072 and AA7072 + AA7075 in methanol liquid and presented simultaneous solutions. The significant outcomes are as follows:

- Momentum and thermal distributions are increasing functions of *n*.
- Flow field is diminished by magnetic field parameter, *M* and a reverse trend is observed for the temperature field.
- The hike in wall thickness parameter results in a lessening in the flow and energy fields.
- The impact of Lorentz force is less on hybrid nanofluid when equated with nanofluid.
- The rate of thermal transport of the hybrid nanofluid is higher than the nanofluid.
- Wall thickness parameter regulates the Nusselt number for both the nanoliquids.
- The major application of the present study can be found in aerospace manufacturing industries.
